# Conditioning Individual Mosquitoes to an Odor: Sex, Source, and Time

**DOI:** 10.1371/journal.pone.0024218

**Published:** 2011-08-26

**Authors:** Michelle R. Sanford, Jeffery K. Tomberlin

**Affiliations:** Department of Entomology, Texas A&M University, College Station, Texas, United States of America; Freie Universitaet Berlin, Germany

## Abstract

Olfactory conditioning of mosquitoes may have important implications for vector-pathogen-host dynamics. If mosquitoes learn about specific host attributes associated with pathogen infection, it may help to explain the heterogeneity of biting and disease patterns observed in the field. Sugar-feeding is a requirement for survival in both male and female mosquitoes. It provides a starting point for learning research in mosquitoes that avoids the confounding factors associated with the observer being a potential blood-host and has the capability to address certain areas of close-range mosquito learning behavior that have not previously been described. This study was designed to investigate the ability of the southern house mosquito, *Culex quinquefasciatus* Say to associate odor with a sugar-meal with emphasis on important experimental considerations of mosquito age (1.2 d old and 3–5 d old), sex (male and female), source (laboratory and wild), and the time between conditioning and testing (<5 min, 1 hr, 2.5 hr, 5 hr, 10 hr, and 24 hr). Mosquitoes were individually conditioned to an odor across these different experimental conditions. Details of the conditioning protocol are presented as well as the use of binary logistic regression to analyze the complex dataset generated from this experimental design. The results suggest that each of the experimental factors may be important in different ways. Both the source of the mosquitoes and sex of the mosquitoes had significant effects on conditioned responses. The largest effect on conditioning was observed in the lack of positive response following conditioning for females aged 3–5 d derived from a long established colony. Overall, this study provides a method for conditioning experiments involving individual mosquitoes at close range and provides for future discussion of the relevance and broader questions that can be asked of olfactory conditioning in mosquitoes.

## Introduction

As small animals with limitations on vision and mobility, insects rely heavily on olfactory information that can be carried through air currents, providing critical information about habitat, food, mate and host associated cues in a complex environment without the need for direct assessment [1]. Thus, the ability to associate an odor with a resource confers significant advantages with respect to resource utilization and local adaptation [2], [3], [4]. Learning about odors associated with specific resources can reduce search times and provide the insect with the plasticity required from generation to generation in rapidly changing environments [5]and is considered a ubiquitous property of insects [4].

Associative learning research with insects has centered around four model species and has relied heavily on odor associated conditioning experiments. The vinegar fly, *Drosophila melanogaster* Meigen (Diptera: Drosophilidae), is a widely used model organism for odor-based associative learning that utilizes a group training method. Large numbers of individuals, ranging from 40–200 [6], [7], [8], [9], are simultaneously conditioned to an odor using an aversion method of an electrified screen [9], [10], which has allowed researchers to explore the genetic basis of olfactory conditioning in *D*. *melanogaster* with genetic drop out mutants [10], [11], [12], [13]. The proboscis extension reflex (PER) of the honey bee - *Apis mellifera* L. (Hymenoptera: Apidae), provides an unambiguous movement of the bee's proboscis in response to a sugar solution that can be observed and scored by the observer [14]. A pairing of the sugar solution and a puffed air stream containing the odor to which the bee is being conditioned provide the framework for individual honey bee conditioning [15], [16]. Tobacco horn worm moths - *Manduca sexta* (L.) (Lepidoptera: Sphingidae), presented with a sugar solution activate the cibarial pump, an organ used to extract nectar solutions from their source and into the insect via the long proboscis, which can be conditioned to an odor exposure [17]. Moths, in general, may possess a high level of olfactory resolution due to their dependence on complex and discrete pheromone structures and blends that impose reproductive isolation mechanisms for closely related species [18], [19]. The capabilities of *Microplitis croceipes* Cresson (Hymenoptera: Braconidae), for conditioning and detection of odors are discernable to the molecular level [20] and capable of distinguishing novel odors from within an odor blend This parasitoid wasp has been developed as a “bio-sensor” due to its high level of ability for detecting specific components of odor blends [21], [22]. In this insect model, as opposed to the other models, the conditionable response represents a suite of reflexive behaviors rather than a single distinct reflex consisting of either an oviposition preparation response [23] or a food acceptance response [24].

Ecologically similar to *M*. *croceipes*, mosquitoes (Diptera: Culicidae) must discriminate among various competing cues when searching for blood-meal hosts, carbohydrate sources and oviposition sites at various points during their life history. The significance of associative learning in mosquitoes is perhaps most evident in the potential that such associations may have on the vector-host relationship [25]. Those female mosquitoes that vector pathogens have the largest impact on pathogen transmission when they seek out and obtain their next blood-meal and transfer the pathogen to the next host. It has been suggested that preferential feeding through a conditioning mechanism on the least defended, most susceptible individuals, in the population (the infected population) may affect the assumptions of biting frequency often used in vector disease modeling [25]. Heterogeneity of biting, in which mosquitoes restrict their blood-feeding behavior to certain individuals within a host-population rather than randomly distributing bites among all potential hosts (homogeneous biting), has been linked to the heterogeneity observed in patterns of disease prevalence [26], [27]. The pattern is striking, such that only 20% of individuals are burdened with 80% of the *Plasmodium falciparum* infection as observed in African countries [27]. Though other factors such as host susceptibility to infection may play a role, the ability for vector's to learn about their hosts may play a role in describing the causes underlying heterogeneous biting which have not been thoroughly explored.

Patterns of host and site fidelity have been observed in multiple mosquito species. Site fidelity , in which mosquitoes returned to a home range has been documented for Anopheles *funestus* Giles [28], *A. farauti* Laveran [29] and *A. arabiensis* Patton [30]. All of these studies used capture-mark-release-recapture to observe mosquitoes returning to their village or place of initial capture. The mechanism suggested to account for the return of mosquitoes was olfactory though other factors such as landmark learning were not ruled out. In *A. balabacencis* Baisas, a higher percentage of mosquitoes that had taken blood from either a buffalo or a human returned to the same host-type at the next blood-meal opportunity [31]. Using a similar method significantly more *Culex* spp. mosquitoes that had successfully fed on either a pig or a cow returned to the same host-type at the next opportunity and when individual mosquitoes reared from the eggs obtained from this blood-feeding were tested for innate preference they lacked the preference exhibited by the parental generation [32]. Thus, this result strongly supported that conditioning had occurred.

For the experiments comprising this study we used the southern house mosquito, *Culex quinquefasciatus* Say. It is a cosmopolitan mosquito with vector potential for both viruses [33] and nematodes [34], [35], which suggests that it has a wide range of potential hosts but demonstrates some level of fidelity if it is considered a significant vector because repetitive blood-meals are required for pathogen transmission. Sugar-feeding is an essential component of mosquito life history exercised by both males and females [36] which, when used as a conditioning reward, avoids the confounding factors associated with the observer in the study being a potential blood-meal source [37]. It also allows for the examination of male mosquitoes which may have potential value in the development and use of toxic-sugar-bait technologies (TSB) [38], [39]. If mosquitoes return to flower odors or types that have yielded a successful sugar-meal in the past they may be more likely to return to this flower-odor combination in the future and hence increase the effectiveness of TSB. In addition, male mosquitoes are often overlooked in the study of mosquitoes, most likely due to their lack of blood-feeding, however the value of male mosquito behavioral data may be more important to control technologies such as transgenic mosquito introduction where the introduced mosquito (male or female) must interact successfully with the wild population [40], [41]. In this study, we conditioned mosquitoes to an odor associated with a sugar-meal to develop a more detailed accounting of mosquito learning abilities as compared to other insect model systems. In this respect, we examined the behaviors associated with close-range feeding behavior.

Associative learning of odors by mosquitoes has been doubted by authors in previous work [37], [42] and has been documented by others [43], [44]. Yet the studies examining odor-based conditioning have thus far, failed to demonstrate several key aspects of conditioning, present in other model insect systems, which may provide a more convincing demonstration of associative learning of odors in mosquitoes in the following areas. Unlike studies that have examined the more long range upwind flight behaviors of mosquitoes in response to conditioning [42], [44] the response of the mosquito at close range has not been discretely described but rather assumed in previous literature examining close-range conditioned behavior [43]. The assessment of the odors used in conditioning mosquitoes at close range has also not yet been determined [43]. The ability to select for certain learning capabilities during laboratory colonization has been demonstrated in *D*. *melanogaster* [45], [46] and thus becomes a consideration in the use of colonized mosquitoes for learning experiments. And finally the determination of whether the conditioned close-range responses persist for ecologically relevant time periods as mosquitoes may sugar-feed perhaps once every 24 hr [36]. In this study we examined the following: the presence of an unconditioned response reflecting exposure to sugar, an evaluation of the naïve response to the unconditioned stimulus, conditioned responses of males and females both colony derived and wild, that conditioned responses can persist for ecologically relevant times, and that experimental design can consist of individual level conditioning and robust statistical analysis with binary logistic regression.

## Results

### The unconditioned response of *Culex quinquefasciatus* to sugar

When presented with a sugar solution within the confines of a colony cage both male and female mosquitoes display a patterned search response. Mosquitoes actively walk along the bottom of the colony cage near the sugar source moving their proboscis in a probing manner. This search behavior has also been observed in the field by Haeger [47] who observed the mosquito *Aedes taeniorhynchus* (Wiedemann) “crawling and probing” extra-floral nectaries on a tree branch. Video S1 illustrates this behavior by several male mosquitoes exposed to a 10% sucrose solution in the absence of introduced odor. The mosquitoes use the proboscis to search for sugar. In Video S2 mosquitoes can be seen feeding on a cotton sugar wick consisting of a 10% sucrose solution that has been colored with green food coloring to facilitate visualization of when the mosquitoes have fed two male mosquitoes (indicated by a yellow and a blue arrow) were observed engaged in the probing-walking behavior on the cage floor. A resting female can be seen on the side of the vial filled with the green sugar solution and not performing this search behavior (indicated by a purple arrow). The probing-walking response is also a directed movement such that the mosquito continues this behavior in the direction of the sugar source. The mosquitoes in the video are most likely responding to small droplets of sugar that have been expelled from the abdomens of feeding mosquitoes as they concentrate the sugar in their crop [36] rather than to any olfactory cue present in the sugar solution (none was added). This response is also present in mosquitoes that have been deprived of sugar for 24 hr, with the search pattern directed at corpses on the bottom of the colony cage (Video S3).

In addition to the response observed on artificial sugar sources without introduced odors, the response can be observed with the presentation of flowers. In Video S4 male and female mosquitoes can be seen using this probing-walking response in search for nectar directly on a picked *Lantana* sp. (Lamiales: Verbenaceae) flower. The probing-walking response (PWR) is a close-range response and represents the unconditioned response to which the mosquitoes were conditioned in further experiments.

### Sugar-feeding Time

Understanding the amount of time necessary for the mosquito to procure a full sugar meal is critical to determining the length of total conditioning events. In this experiment, time intervals between conditioning and testing ranged from <5 min to 24 hr thus the total conditioning time in which the mosquito was allowed to feed needed to be short enough in length that the mosquito retained the drive to sugar-seek after conditioning. Mosquitoes were fed for three 10 sec conditioning time periods in the protocol which was suggested to be sufficient for conditioning by the only previous study to examine individual level conditioning [43].

**Figure 1:**
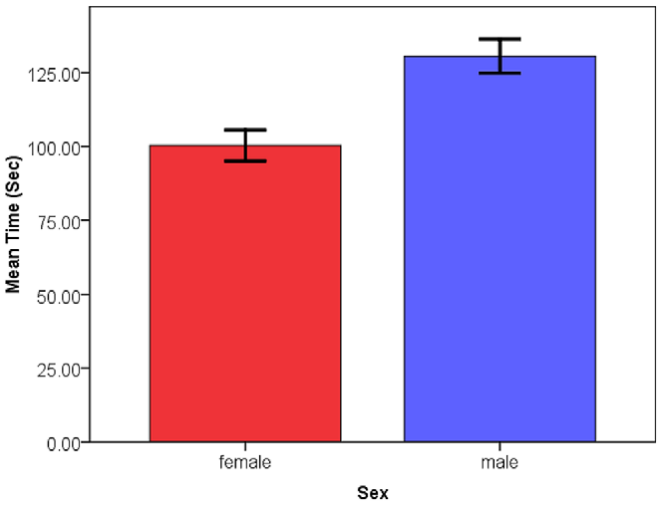
Mean sugar-feeding time of males and females. Mean (+/- SE) sugar-feeding time (s) for female (n = 55) and male (n = 41) *Culex quinquefasciatus*.

**Figure 2:**
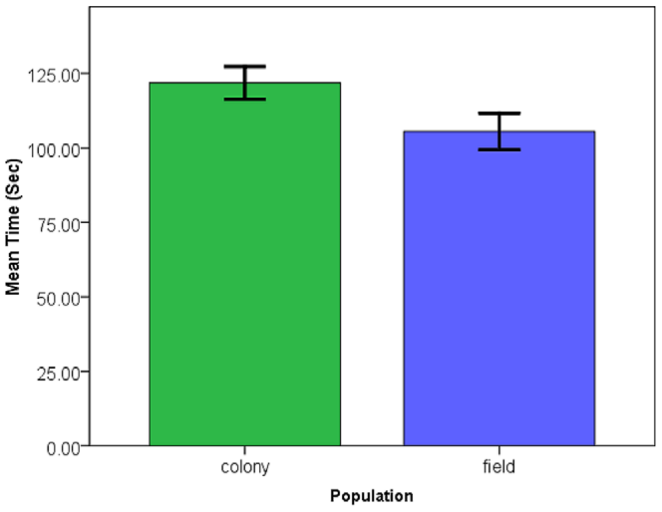
Mean sugar-feeding time from different sources. Mean (+/- SE) sugar-feeding time (s) for laboratory colony (n = 50) and wild collected (n = 46) *Culex quinquefasciatus*.

**Figure 3:**
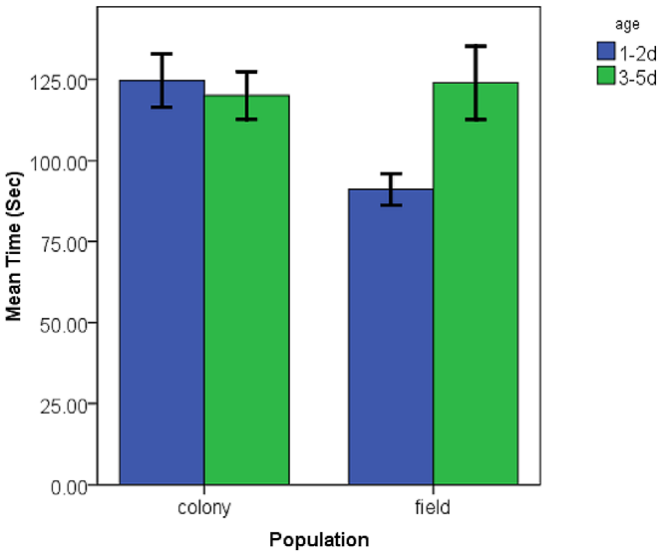
Mean sugar-feeding time: source by sex. Mean (+/- SE) sugar-feeding time (s) for laboratory colony and wild collected *Culex quinquefasciatus* by sex (colony: n = 30 females, 25 males; wild: n = 20 females, 21 males).

Sugar-feeding time for females was generally shorter than that for males (Figure 1) with a mean of 100.3 sec for females while males took 151.4 sec on average overall. The final model for the ANOVA of sugar-feeding time is presented in Table 1. Significant factors included the population of the mosquito with colony mosquitoes taking significantly longer sugar-meals (Table 1; Figure 2), the sex of the mosquito where males took significantly longer to take a sugar-meal (Table 1; Figure 1), and there was a significant interaction between population and age of the mosquito with 3–5 d old mosquitoes taking a longer time to sugar-feed than 1–2 d old mosquitoes from the field collected population (Table 1; Figure 3). The amount of time until satiation is well below the time used for conditioning (30 sec) thus the mosquitoes were assumed to have maintained the need to search for a sugar-meal after conditioning in order to assess the PWR at testing.

**Table 1:** Full factorial analysis of variance for sugar-feeding time based.

Factor	d.f.	F-statistic	*P*-value
**Population**	1	5.744	0.019*
**Age**	1	2.331	0.130
**Sex**	1	19.545	<0.001*
**Population × Age**	1	5.353	0.023*
**Population × Sex**	1	0.919	0.340
**Age × Sex**	1	0.834	0.364
**Population × Age × Sex**	1	0.006	0.938
**Error**	88	-	-

*Indicates significance observed at the α = 0.05 level.

The factors of mosquito population (laboratory or field collected), age of the mosquito (1–2 d old or 3–5 d old), and sex of the mosquito (male or female) were analyzed with a full factorial analysis of variance.

### Evaluation of the conditioned response

**Figure 4:**
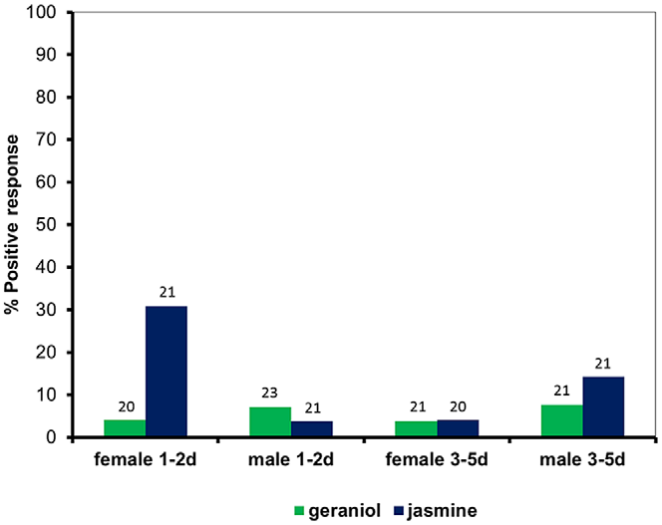
Innate positive response to the experimental odors. Percent positive response to the odor of jasmine flavor extract and geraniol by naïve male and female *Culex quinquefasciatus* aged 1–2 d and 3–5 d old. Sample sizes are indicated on the figure.

The target and non-target odors were evaluated for their ability to elicit the PWR by presentation of the odor as in the conditioning protocol followed by observation of behavior. Laboratory mosquitoes aged 1–2 d, which had never had access to sugar, and mosquitoes aged 3–5 d that had been deprived of sugar for 24 hr were offered an empty pipette swabbed with either the target - jasmine or non-target - geraniol, demonstrated different levels of PWR upon exposure (Figure 4). Naïve response to the odor of geraniol never exceeded 10% from either of the age groups regardless of sex. Younger females (1–2 d old) exhibited the strongest response with approximately 30% (n = 21) of those exposed exhibiting the PWR. Jasmine odor did not have the same effect on males at 1–2 d old (n = 21) or on older females (n = 20), however approximately 15% of older males (3–5 d old; n = 21) responded with the PWR.

### Conditioning of *Culex quinquefasciatus*

**Figure 5:**
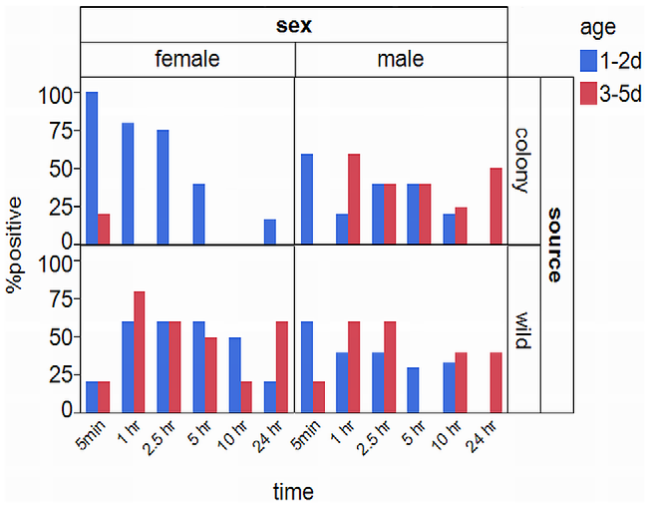
Positive response to the target odor after conditioning. Percent positive response to the target odor following conditioning by female and male mosquitoes from laboratory colony and field populations at six different time intervals between conditioning and testing by age.

The percent positive response to the target odor following conditioning for each population, age and sex by time period between conditioning and testing is plotted in Figure 5 (responses to the non-target and blank tests are provided in the Supporting Information as Figure S2 and Figure S3 respectively). Overall, the median positive response to the target odor following conditioning was 40%. In general the trends present in positive response to the target odor are not striking. There is one exception for1–2 d old female laboratory derived mosquitoes where there was a steady reduction in percent positive response to the target odor with time. In contrast, the only positive responses to the target odor for 3–5 d old mosquitoes were at the shortest time period of less than 5 min (Figure 5). There was also a lack of responses for 1–2 d old male mosquitoes from the field-collected population at 24 hr, which is due to the fact that approximately 95% of them died before testing could be conducted.

Table 2 displays the final model after both manual removal of non-significant interaction terms and backward stepwise variable selection using maximum -2log-likelihood. Model selection ended at step 13 had a -2 log-likelihood of 397.488 and Hosemer-Lemeshow statistics indicated the model was significant (χ^2^ = 14.290, d.f. = 7, *P* = 0.046). Logistic regression results differ from least squares regression in that the model compares the binary outcomes between categories and for groups with more than two categories a dummy variable is created to compare within each group. This provides log odds (or odds ratios) and generates predicted probabilities for the categories. The odds ratios and Wald statistics for each factor in the model are presented in Table 2 as well.

**Table 2:** Final model for the modified factorial binary logistic regression analysis of mosquito response to conditioning.

Factor	Log Odds (β)	Wald Statistic	d.f.	*P*-value	Ratio
**Age (1–2d)**	0.487	3.011	1	0.083	1.627
**Test**	-	88.898	2	<0.001*	0.080
**Test (blank)**	-2.528	53.138	1	<0.001*	-
**Test (non-target)**	-3.322	48.121	1	<0.001*	0.036
**Source (lab) × Sex (female)**	-2.912	7.931	1	0.005*	0.54
**Source (lab) × Age (1–2d) × Sex (female)**	3.533	9.513	1	0.002*	34.252
**Source (lab) × Age (1–2d) × Time × Sex (female)**	0.000	4.445	1	0.035*	1.000

*Indicates significance observed at the α = 0.05 level.

This model was based on the factors of age of the mosquito (1–2 d old or 3–5 d old), the test the mosquito was administered (target, non-target, or blank odor pipette), the interaction between the source the mosquito was derived from (laboratory or field collected) by the sex of the mosquito, the interaction between population, age of the mosquito and sex of the mosquito and the interaction between the source, age, sex and time interval between conditioning and testing.

#### Success of Conditioning

**Figure 6:**
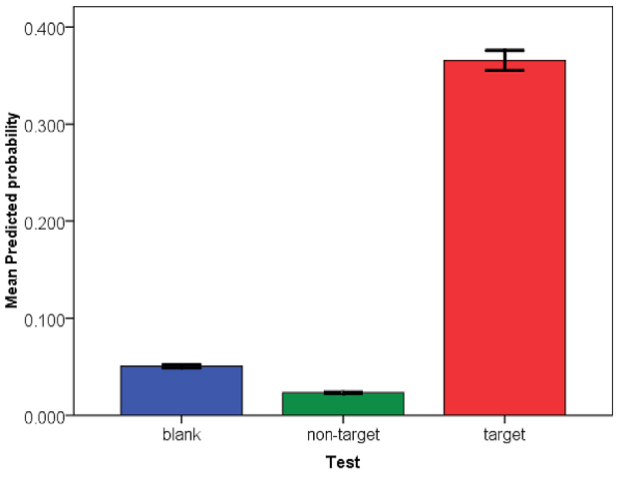
Predicted probabilities of positive response following conditioning. Mean (+/- SE) predicted probabilities of positive response generated by the logistic regression model for mosquitoes offered an un-scented blank pipette, the non-target odor or the target odor; including males and females aged 1–2 d and 3–5 d from both the laboratory colony and wild collected material.

The significance of the test factor in the model is important, as it indicated that there was a significant difference in odds of responding to the target odor when compared to the non-target or blank (Table 2). This result is confirmed by examining Figure 6, which displays the mean predicted probabilities for positive response to the blank, non-target, and target odors for all combinations of the factors examined in this study. There were significantly higher odds of positive response to the target odor regardless of any of the other factors. A Kruskal-Wallis analysis of variance followed by individual Mann-Whitney U tests confirms that the mean predicted probabilities are in fact significantly different (K-W χ^2^ = 400.019, d.f.  = 2, *P*<0.001; M-W U (blank vs. non-target) = 7197.000, *P*<0.001; M-W U (blank vs. target)  = 5782.00, *P*<0.001; M-W U (non-target vs. target)  = 2730.000, *P*<0001). From this result, we can infer that conditioning was successful.

#### Mosquito Age

The only factor that was not significant on its own in the model was the age of the mosquitoes. However, the odds ratio suggests that there were 1.627 higher odds that a mosquito from the 1–2 d old age group would respond positively (Table 2).

#### Mosquito Population and Sex

**Figure 7:**
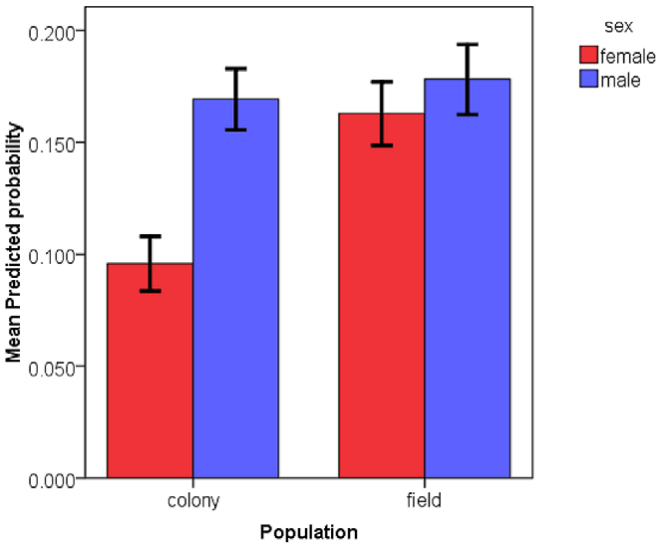
Predicted probabilities of male and female mosquitoes after conditioning. Mean (+/- SE) predicted probabilities of positive response generated by the logistic regression model for female and male mosquitoes derived from either the laboratory colony or field collected populations.

**Figure 8:**
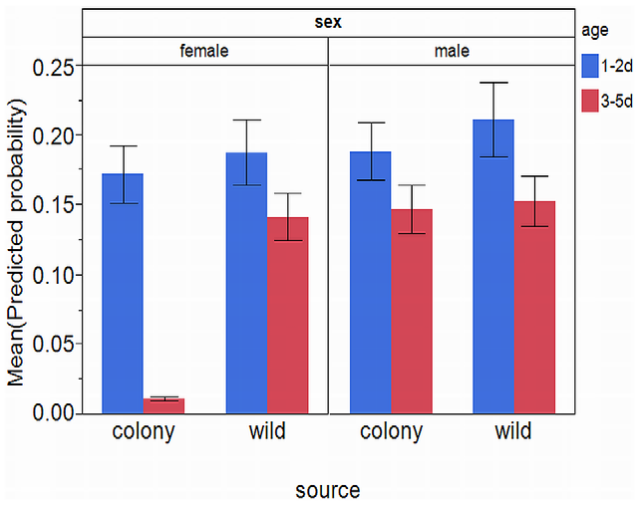
Predicted probabilities of conditioned mosquitoes across sex, source, and age. Mean (+/- SE) predicted probabilities of positive response generated by the logistic regression model for male and female mosquitoes from laboratory colony and field collected populations by age.

The interaction between the population that the mosquitoes were derived from and the sex of the mosquitoes was a significant factor in the overall model (Table 2). There were -2.912 odds that a female mosquito from the laboratory colony would respond positively which was supported by the mean predicted probabilities plotted in Figure 7. There was a significantly lower mean predicted probability that females from the laboratory colony population would respond positively (Figure 7; M-W U = 9859.000, *P*<0.001). A significant interaction between the population the mosquitoes were derived from, the age of the mosquitoes, and their sex was also determined (Table 2). The logistic regression model indicates there were 3.533 higher odds that a female mosquito from the laboratory colony, aged 1–2 d old would respond positively. A plot of the mean predicted probabilities illustrates this relationship (Fig. 8). This is likely attributable to a surprising lack of positive responses from laboratory colony females aged 3–5 d old (Figure 5). The mean predicted probabilities of 1–2 d old mosquitoes of all test categories are significantly higher than those of 3–5 d old mosquitoes (M-W U (females, laboratory colony)  = 600.000, *P*<0.001; M-W U (females, field collected)  = 2204.000, *P*<0.001; M-W U (males, laboratory colony)  = 2724.000, *P*<0.001; M-W U (males, field collected)  = 1602.000, *P* = 0.001). We can thus infer that mosquitoes aged 1–2 d old regardless of population derivation had a higher probability of positive response following conditioning.

#### Duration of the Conditioned Response

**Figure 9:**
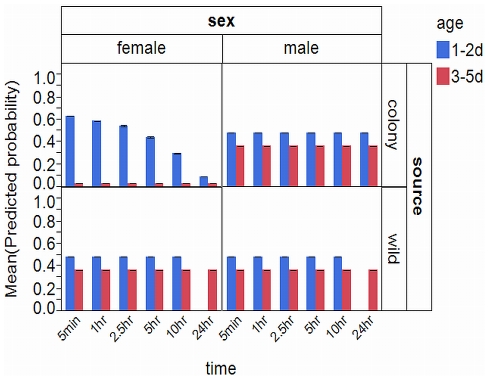
Predicted probabilities of conditioned mosquitoes across sex, source, age and time. Mean (+/- SE) predicted probabilities of positive response generated by the logistic regression model for female and male mosquitoes from laboratory colony and wild populations for six different time intervals between conditioning and testing by age.

The only significant factor in the model relating to the time between conditioning and testing, was the interaction between the population the mosquito was derived from, the age of the mosquito, the sex of the mosquito and the time between conditioning and testing (Table 2). The model indicates that there were significantly higher odds that a female mosquito from the laboratory colony, aged 1–2 d old would respond positively based on the time between conditioning and testing which is illustrated in the plot of mean predicted probabilities (Figure 9) which represents the trend observed in the actual data (Figure 5). The mean predicted probabilities plot does not show significant differences among any of the other groups but does display an unusually high predicted probability for 1–2 d old males from the field-collected mosquitoes at the 10 hr time interval (Figure 9). This high value does not match the observed data (Figure 5) but perhaps is an artifact of the missing 24 hr male data for this population and age. Although not a significant comparison, there does appear to be a trend of higher mean predicted probabilities for the longest time period among mosquitoes in the 3–5 d old age group with the only exception being the females from the laboratory colony population (Figure 9). The overall trend can also be observed that mean predicted probabilities are higher on average for all other time periods with available data except for the longest time period (Figure 9), however this is not a significant trend in the model generated predicted probabilities.

## Discussion

The mosquito, *Culex quinquefasciatus*, displays a patterned, close-range, behavioral response to sugar exposure that can be observed and conditioned. Both male and female mosquitoes can be conditioned with varying degrees of success over ecologically relevant times. Laboratory colonization of this mosquito species may have effects on the maintenance of associative odor learning in females. This study has demonstrated and defined some key aspects of olfactory conditioning in mosquitoes that allow us to more easily relate it to established model insect systems.

### Defining the unconditioned response

A fundamental feature of classical conditioning experiments is the establishment of the defined unconditioned response. In this study, we demonstrated that *C. quinquefasciatus* displays a patterned search response following exposure to sugar when observed in a physiological state associated with 24 hr of sugar deprivation. We term this response the probing-walking response (PWR) which has also been observed in other species of mosquito, including *A. taeniorhynchus* [47], *Anopheles cracens* Sallum & Peyton and *Anopheles minimus* Theobald [48]. Defining the PWR with respect to sugar-feeding provides an established behavioral pattern that can be reproduced and observed in future experiments.

### Sugar-feeding Times

Sugar-feeding times were initially collected to ensure that the length of the conditioning protocol was short enough that the mosquito maintained motivation to continue sugar-seeking for the testing portion of the protocol. Males took a significantly longer time to sugar-feed than females. One possible explanation is that males are not under the same level of pressure to feed fast that females are when blood-feeding [49] so they can essentially take their time feeding. Another possible explanation is that the females are filling the crop with the sucrose solution rather than the midgut [50] and this could be a shorter process. It is also interesting to note that younger mosquitoes taking their first sugar-meal from the field-collected population took shorter sugar-meals. A potential explanation for this observation may be that by 3–5 d of age the older mosquitoes had experience with 10% sucrose in the cage and been conditioned to anticipate access to sugar. Thus, they took a longer time to feed to satiation. Differences in volume of the meal may explain the variation observed, but it seems counterintuitive that the mosquitoes would take a smaller sugar-meal as their first meal however is may be a reflection of favorable larval nutrition [51].

### Neutrality of Odor

One difficulty present in conditioning experiments involving odors is how to determine that an odor has been detected but is a neutral stimulus, such that is it not an attractant or a repellent. The high level of interest in mosquito attractants and repellents has led to the development of indices of attraction and repellency to both host-seeking associated odors [52], [53] and oviposition substrates [54]. Thus the literature tends to identify these chemicals as belonging to one of these groups based on the endpoint of a behavioral sequence It has been shown in *Drosophila* that olfactory conditioning changes the pattern of stimulated brain areas, such that an odor detected and identified by a certain pattern and activation of antennal lobe glomeruli is different following conditioning [55]. This suggests that the odor was not entirely novel, at least at the receptor level and it suggests that conditioning is occurring at higher brain centers. Mosquitoes may display some behavioral response to odors initially and fine tune their response to specific odors through repeated exposure paired with a meaningful resource [44] much like what has been proposed for parasitoid wasps [5].

### Conditioning of *C. quinquefasciatus*

Conditioning of *C. quinquefasciatus* was successfully accomplished using a close-range individual-based approach. Significantly higher predicted probabilities of a positive response to the target odor occurred in mosquitoes that had been conditioned to that odor. Conditioned responses were observed in this mosquito over several different experimental conditions.

#### Sex Specific Differences

One of the common behavioral traits of male and female mosquitoes is their propensity to sugar-feed [36], [56]. In this study both male and female mosquitoes were found to be capable of olfactory-based conditioning to a sugar-meal. The significance of the interaction between the sex of the mosquito, the age of the mosquito, and the population it was derived from illustrate one of the few sex-based differences observed for the conditioning of the mosquitoes in this study.

The sex of the mosquitoes had the biggest impact on whether the mosquitoes would demonstrate a positive response after conditioning if they were 3–5d old and derived from the laboratory colony (Figure 8). This observed effect may have been due to the natural shift in females from carbohydrate-seeking behavior to blood-seeking, which is complicated by the fact that the observer for the experiments is a potential host. Working on associative learning of host-associated odors can lead to complications due to the observer/experimenter being a potential contaminating source of host odor [37] and is one reason why this experiment examined associating sugar-meals and odors. However, by 3 d of age female *Culex tarsalis* Coquillet can start to take blood-meals and develop eggs under optimal field conditions [57] and at 5 d of age the laboratory colony of *C. quinquefasciatus* is offered a blood-meal for normal egg production and colony maintenance (Texas A&M University AUP#2007-162). Thus at 3–5 d old the females from the colony may have already shifted to blood-seeking and suggests that the colony has been selected for a 5 d blood-feeding cycle. This is supported by the lack of this observation in the field derived mosquitoes at 3–5 d old (Figure 8) and supports the importance of consideration of starting material for experiments with implications beyond the laboratory.

#### Sugar-feeding Experience

The results of this experiment suggest that mosquitoes conditioned to a sugar-associated odor at a younger age (1–2 d old) show a higher probability of a positive response following conditioning. Considering this result in the context of its ecological relevance the association made between the resource and odor at an early life stage may be the most significant if the association is adaptive. Learning is adaptive if it increases within lifetime fitness in a habitat with higher inter-generational variation than intra-generational variation [2]. It seems intuitive that learning at an early life stage can be maintained throughout adult life in a short-lived organism like a mosquito.

An additional consideration with respect to the age of the mosquito is its ability to form memory, which can be significantly affected by age. In *Drosophila*, flies that are older than 20 d have significantly reduced long-term memory formation [58], [59]. Long-term memory consists of memory that spans several days [6] and would be the most informative type of adaptive memory to rely upon in a habitat for the span of the mosquito's adult life. Thus learning as a young adult may be more likely to result in long-term memory formation and may have the most significant impact on lifetime fitness.

#### Source Material Origin

The origin of the mosquitoes in this experiment had observable effects on the probability of positive response following conditioning at longer times after training. Specifically this was evident for females aged 3–5 d old, from the laboratory colony which showed little to no evidence of conditioning beyond the 5 min time interval. In *Drosophila*, Mery and Kawecki [60] suggested that selection for traits associated with learning are broader than just a simple associative task. They showed that learning itself is under selection and the selection pressure is in the context of ecologically-relevant information. If there is a cost associated with maintaining learning ability [45], laboratory colony derived females are not under selection pressure to maintain it. All the resources a mosquito needs are within the confines of the colony cage. However, if this is the case, then it might be expected that males would also lose this ability, and this was not observed in the current study. Nevertheless, it emphasizes the need to use source material relevant to the application of the data, such that if the data are to be applied to a field system they should be derived from a field population if possible.

#### Duration of the Conditioned Response

No significant effect was observed for the duration of the conditioned response in *C. quinquefasciatus* using the current analysis. The trend that was observed suggested younger mosquitoes displayed approximately the same level of predicted probability of positive response or slightly higher than the older mosquitoes at all time periods except at the longest time period of 24 hr (except for laboratory colony derived females). Though not significant, it suggests that positive response may be higher for the longest time interval in older mosquitoes. However, this result may be an artifact of the missing 1–2 d old male field derived mosquitoes, which died before testing. This experimental design is limited by the fact that mosquitoes may not live without sugar for periods of time longer than 24 hr (Figure S1). Thus in order to maximize the effort required to condition each individual 24 hr was selected in this experiment as the longest time interval that could be reasonably accomplished without significant mortality before testing. To evaluate learning in a more ecological framework experimental design changes could be implemented to encompass conditioning over longer time intervals with the expectation that long-term memory formation is occurring [6], [61] but further experiments will need to be completed with respect to how long the inter-trial interval can be to result in a persistent conditioned response.

#### Experimental Design

An experimental design that allows for individual level testing and evaluation can be accomplished with this mosquito species. It allows for investigation of very specific experimental conditions but for the same reason it can lead to very difficult to attain levels of individual replication requirements for powerful statistical evaluation and may not capture the complexity of a natural learning experience. Individual mosquitoes can be difficult to condition and evaluate within restricted time limits and the reduction of researcher bias and location bias by limiting experiments to a single individual in a single location further reduces the potential for very large sample sizes. Preliminary group conditioning of mosquitoes has been attempted (Sanford, Unpublished Data) but has not yet been fully evaluated with respect to the level of conditioning that can be expected with a group technique.

Evaluation of responses with a strict binary outcome has both advantages and disadvantages. One advantage is the unambiguous outcome of each individual for analysis, which is not based on a percent response that reflects the outcome of multiple individuals. Another advantage is the ability to use binary logistic regression models that can incorporate many of the interaction terms that are associated with interpretation of General Linear Models [62], [63]. The use of a binary logistic regression model may also be the biggest disadvantage of the experimental design as it is a difficult analysis to interpret. Odds ratios do not provide the same intuitive result that other regression models provide and thus can leave one with the sense that there is a high level of reliance on the statistical analysis rather than the raw data. However, in this study the raw data (Figure 5) reflect the predicted probabilities generated from the binary logistic regression model (Figure 9). Suggesting, at least overall, that the raw data used to generate the model created an accurate representation in terms of log odds and predicted probabilities.

### Considerations

The study presented here represents a significant improvement over the first documentation of appetitive olfactory-based associative learning in this species [43]. However, there remain areas where the experimental design could benefit from further improvement. The basis of the conditioning and testing of the mosquitoes, being individually based requires a substantial time commitment limited by the lifetime of the individual mosquito. Thus, a trade-off is generated between the sample sizes required to generate adequate statistical power and the need to balance the experimental design with reciprocal odor pairing. In this study, we made the decision to trade off the reciprocal odor pairing based on literature in other species [24], [64], [65], [66] and our interest in addressing the experimental questions within the time and funds available. This is a limitation of the current study and a point of interest to those designing experiments with the methods described here. Additional considerations should also be given to observer bias and future studies might consider using a blind approach to testing, which may help to mitigate this issue.

### Significance and Future Direction

As compared to previous work in mosquito learning that has focused on conditioning of long range flight-based behaviors [42], [44] the current study has demonstrated that conditioning can be accomplished using the close-range behaviors of *C*. *quinquefasciatus*. Upwind flight consists of multiple behavioral units that are linked to result in attraction to a resource [67], [68]. One might envision that in natural settings over longer distances, the mosquito must integrate multiple information sources for flight and then once close to the resource another set of behaviors must be used to assess, accept and acquire the resource. It seems logical to suggest that because both long-range and close-range behaviors are intimately linked with resource acquisition they are subject to conditioning at different decision points in the process of resource acceptance. The next major step in determining how learning in mosquitoes influences their interactions with hosts and pathogens is to determine to what extent these two aspects of conditioned behavior are linked. If a mosquito is conditioned at close-range, does that confer long-range conditioning? Conversely does conditioning at long-range confer close-range conditioning? The answers to these questions may help to elucidate the importance of close-range conditioned behavior for larger applications such as determining the impact of conditioning on Toxic Sugar Bait control methods [38], [39], wild mosquito population behaviors with respect to home ranges [28], [29], [30] (for sugar- or blood- feeding) and on pathogen transmission [25].

## Materials and Methods

### Experimental Design

This experiment was designed to demonstrate and compare conditioning of mosquitoes at their first sugar-feeding experience (1–2 d old) and those 3–5 d of age, of both sexes, from both wild and colony derived populations for increasing periods of time up to those considered ecologically relevant. Ecologically relevant times were defined as the period of time between mating swarm events in the field (typically dusk and dawn transitions, approximately 10–24 hr) when mosquitoes must sugar-feed to acquire the energy for the next mate swarming event [36]. The time points that were used for testing were: <5 min, 1 hr, 2.5 hr, 5 hr, 10 hr, and 24 hr. The average amount of time that male and female mosquitoes lived after receiving either a full sugar-meal, as in the sugar-feeding time evaluation, or a partial sugar-meal, as they received in the conditioning protocol exceeded 24 hr (Figure S1). A minimum of 15 males and females from each age and population were conditioned for each test interval (N = 720). Following the completion of the entire time series an additional 30 males and females for each time period were conditioned to confirm the observed trends with a larger within time sample size using the laboratory colony derived population.

### Mosquitoes

Two sources of *Culex quinquefasciatus* Say were used in this study representing laboratory colony and field collected populations. The laboratory colony consisted of mosquitoes originally derived from material collected in Gainesville, FL, USA in 1992 but obtained from the Center for Medical, Agricultural, and Veterinary Entomology (CMAVE), USDA-ARS, Gainesville, FL, USA in January 2009 for colony establishment at Texas A&M University, College Station, TX, USA. Larvae were reared using a standard method of two egg rafts per liter of deionized water in white enamel pans on a diet consisting of a ground Tetramin® Tropical Flakes (Tetra Holding (US) Inc., Blacksburg, VA, USA) slurry consisting of 3 parts ground Tetramin® to 1 part deionized water.

The field-collected population was obtained by collecting egg rafts at a single field site location over the course of the experiment (April – September 2009) adjacent to a wooded drainage canal in College Station, TX, USA (approximately: 30°36'47 N, 96°19'26 W). Egg rafts were collected using a modified Reiter media [69] consisting of 75 gm Bermuda grass (Kaytee Natural Bermuda Grass, KAYTEE Products, Inc., Chilton, WI, USA) and 4.6 gm dried active baker's yeast (MP Biomedicals, Inc., Solon, OH, USA) in 18.92 l of tap water fermented in sealed 5-gallon (18.93 l) buckets for approximately seven days. In the evening oviposition media was deployed in a 11.4 l white dish pan (Sterilite Corporation, Townsend, MA, USA) filled approximately half-full (∼5.7 l) and egg rafts were collected the following morning. Egg rafts were collected for each time interval tested so all material was F_0_ generation. Larvae were reared at a density of one egg raft per liter of deionized water to ensure that each egg raft was *C. quinquefasciatus* and samples of 3^rd^ and 4^th^ instar larvae were pulled from developing cohorts so identification could be confirmed. In addition, adult samples were preserved from every field-collected cohort and have been deposited in the Texas A&M University Insect Collection (voucher #682) for future reference.

For both the laboratory colony and the field collected mosquitoes, pupae were collected and mixed before placing approximately 100 individual pupae on the second day of pupation (to ensure collection of male and female pupae) into each of two cages (small Plexiglass cages: 19.5×19.5×19.5 cm). One cage was held without sugar but with access to water for tests involving 1–2 d old mosquitoes and the other cage was given access to a 10% sucrose solution on a soaked cotton wick up to 24 hr before testing of the 3–5 d old mosquito age group. All mosquitoes were maintained in a Rheem Environmental walk-in growth chamber (Ashville, NC, USA) at 25–27°C and approximately 50–70% relative humidity with a 14∶10 L∶D cycle.

### Obtaining the unconditioned response

Male and female *C. quinquefasciatus* were starved for 24 hr at 1–2 or 3–5 d old and exposed to a 10% (w/v) sucrose solution (technical grade sucrose; Sigma-Aldrich, Co., St. Louis, MO, USA) dyed with green food coloring (Apple Green Color, Royallee Brand, Bangkok, Thailand). Coloring of the sugar-meal facilitated observation of when mosquitoes had fed, including whether probing was associated with feeding and whether the probing behavior stopped after feeding. The response of the mosquitoes was observed while they were allowed to sugar-feed and video was collected (Videos S1 and S2). Starved mosquitoes were also observed to display this behavior on corpses on the bottom of the colony cage before the introduction of the sugar solution (Video S3). As soon as this was observed, a few mosquitoes were transferred by petri dish to a dissecting microscope for rapid observation and video was rapidly taken through the eyepiece of the microscope to document the behavior. A *Lantana* sp. flower was picked, and handled with gloved hands, to observe the behavior of the mosquitoes on more natural sugar source (Video S4). All videos were collected with a Canon PowerShot IS-S2 digital camera (Canon USA, Inc., Lake Success, NY, USA) with or without a tripod.

### Mosquito conditioning protocol

#### Sugar-feeding time

The amount of time that male and female mosquitoes sugar-feed was evaluated to ensure that by the end of conditioning the mosquito had not exceeded the time required for a full sugar-meal which would render them without motivation to continue sugar-seeking [36]. This was accomplished by feeding individual mosquitoes a 10% (w/v) technical grade sucrose (Sigma-Aldrich, Co., St. Louis, MO, USA) solution dyed with red food coloring (to enhance visualization of the filling mosquito gut; Strawberry Red Color, Royallee Brand, Bangkok, Thailand; concentration = 12 drops:30 ml solution) from a new 200 µl calibrated micropipette (Drummond Scientific Company, Broomall, PA, USA). The amount of time from the beginning of feeding until the mosquito pulled the proboscis out of the pipette (this was assumed to represent satiation) was recorded for males and females from the laboratory colony and field collections at the 1–2 d and 3–5 d old ages.

#### Odors used

For this experiment, the single target odor of jasmine flavor extract was selected for conditioning (Winners Brand, Bangkok, Thailand). Jasmine flavor extract was chosen as it was used successfully in preliminary work with both *Anopheles minimus* Theobald and *C. quinquefasciatus* in Thailand [48]. The non-target odor selected was geraniol (98%, Sigma-Aldrich, Co. St. Louis, MO, USA) as it has been used in work with *Anopheles cracens* Sallum & Peyton [48] and it is an odor that is known to be detected by the *C. quinquefasciatus* antennae but not considered an attractant [70]. Single target odor experimental designs have successfully been used to demonstrate learning in *M. croceipes* on multiple occasions [24], [64], [65], [66].

#### Conditioning

Mosquitoes were first allowed to acclimate to the laboratory conditions for 30 min before conditioning by moving the cage from incubator into the main laboratory. All conditioning and testing was conducted under a biological safety cabinet (Logic, Purifier© Class II Biological Safety Cabinet, Labconco Corporation, Kansas City, MO, USA) to promote the movement of air and reduce the potential for habituation and odor contamination. Mean airflow speed was 0.47 m/sec where the mosquitoes were located in the middle of the cabinet and 3.48 m/sec on average at the front of the cabinet as measured with an anemometer (Testo 435-1, Testo, Inc., Sparta, NJ, USA). Prior to conditioning, the mosquitoes were each isolated into individual clean glass shell vials (70 mm tall×20.5 mm diameter, 4 dram: 14.787 ml volume) placed on a small square of clean office paper (∼4×4 cm; Discovery® Premium Select, Soporcel North America, Inc., Norwalk, CT, USA). This small piece of paper allowed for rotation of the vial in order to facilitate access to the mosquito proboscis and to standardize the relative positions of the mosquito to the observer and the pipette in the fume hood.

The general conditioning procedure is similar to Tomberlin [43] but there are several key modifications that ensure that conditioning and testing more adequately meet the expectations of demonstrating associative learning. Each mosquito was conditioned by offering them a 200 µl calibrated micropipette (Drummond Scientific Company, Broomall, PA, USA) with the first 1-2 cm filled with a 10% (w/v) technical grade sucrose (Sigma-Aldrich, Co., St. Louis, MO, USA) solution. The outside, distal, ∼1 cm of the pipette was coated with the target odor of jasmine flavor extract at 100% concentration (Winners Brand, Bangkok, Thailand). The odor extract contains volatile chemicals and these were allowed to normalize for a period of approximately 20–30 sec before presentation to the mosquito. The mosquitoes were offered the odor coated pipette for 15 sec by encouraging the mosquito to rest at the bottom of the vial and then lifting the vial approximately 30° and placing the pipette directly onto the mosquito's proboscis (Video S5). This was repeated for a total of three times with a 30 sec resting period between trials. The mosquitoes were then labeled and if testing was to be conducted at a later time a small plug of clean cotton was placed in the top of each vial until testing. The mosquitoes were left in the safety cabinet if the interval between conditioning and testing was 5 hr or less otherwise they were returned to the walk-in growth chamber until testing. Clean nitrile gloves were worn during experiments to reduce contamination of host-associated odors. The videos submitted as Supporting Information were obtained for demonstration purposes so gloves were not always worn.

#### Testing

For each testing category (age 1–2 d and 3–5 d old by laboratory colony and field collected) mosquitoes were tested at each of six different times from conditioning, consisting of less than 5 min, 1 hr, 2.5 hr, 5 hr, 10 hr, and 24 hr. If the conditioning to testing interval was 5 hr or longer the mosquitoes were kept in the walk-in growth chamber until testing. They were then allowed to acclimate to the laboratory conditions as previously described for approximately 30 min before testing.

The mosquito was offered an empty pipette in the same manner as during testing. A new small sheet of paper (∼4 cm×4 cm) was used for testing the mosquitoes so as to prevent contamination of odors potentially absorbed on the paper. It also allowed for the maintenance of the same relationship between the pipette, the mosquito and the observer as during conditioning by allowing for rotation of the mosquito and vial within the fume hood (Videos S6 and S7). The pipette was empty and either swabbed with the target odor (jasmine flavor extract), the non-target odor (geraniol) that the mosquito had never had experience with prior, or it remained blank. As in the conditioning procedure, the ∼1 cm distal portion of the pipette was swabbed with 100% concentration of the odor extract and allowed to normalize (20–30 sec). It was presented to the mosquito for 15 sec just under the shell vial to allow for a response that required the mosquito to move. A positive response to the assay was recorded when the mosquito displayed the PWR behavior (Video S6). Negative responses were recorded when the mosquito moved away from the pipette or did not make a directed movement toward the pipette (Video S7). If the mosquito did not make any detectable movement or response this was also recorded as a negative response. Each mosquito was conditioned and randomly selected to receive only one test to reduce confounding effects of multiple odor exposures and to facilitate statistical analysis.

### Statistical Analysis

For the data on sugar-feeding time, data points more than 210 sec (3.5 min) were dropped as they were identified as being greater than two standard deviations from the mean. The data were also log_10_ transformed for normalization and analyzed using a full factorial analysis of variance (ANOVA) using the general linear model procedure in SPSS 16.0 [71]. The factors in the model were the population of the mosquitoes, the age of the mosquitoes, the sex of the mosquitoes and the interactions among these terms on the time of sugar feeding. The data consisting of the percentages of mosquitoes responding positively to the odors used in the experiment were calculated but no further data manipulations or statistical analyses were performed.

Data from the conditioning portion of the experiment consisted of the categorical variables indicating the age of the mosquitoes, the sex of the mosquitoes, the population from which the mosquitoes were derived, and the time interval between training and testing on the binary outcome variable indicating whether conditioning had been successfully accomplished. For this analysis a full factorial binary logistic regression model was constructed using syntax commands for SPSS 16.0 [71] (S2). Backward stepwise variable selection based on the maximum -2log-likelihood was used. However it never resulted in a significant model, as determined by Hosemer-Lemeshow statistics so interaction terms remaining in the model through the last iteration of variable selection, but that did not contribute significantly to the model, were removed manually and the model selection re-run until a significant model was observed. Predicted probabilities generated by the model were plotted and subjected to non-parametric statistical analysis with either Kruskal-Wallis analysis of variance or Mann-Whitney U tests as appropriate.

All statistical analyses were conducted in SPSS 16.0 [71] with some data manipulations and charts created in Microsoft Excel 2007 (Microsoft, Corp. Redmond, WA, USA) and JMP [72]. Significance for all statistical tests was observed at the α = 0.05 level. The basic SPSS syntax used for the full factorial model is presented in Figure S4.

## Supporting Information

Figure S1 **Mosquito lifespan following partial or full sugar-meal.** Average number of days of life following either a full sugar-meal or a partial sugar-meal, as would be received following the conditioning protocol, for laboratory colony derived male and female *Culex quinquefasciatus*. Letters indicate a significant difference between treatments as evaluated with ANOVA at α = 0.05 level.
(TIF)

Figure S2 **Percent positive response to the non-target odor (geraniol).** Raw percent positive response data for mosquitoes tested to the non-target odor of geraniol. Data are presented for male and female *Culex quinquefasciatus* adults conditioned to jasmine odor extract from laboratory colony and field-collected material aged 1–2 d or 3–5 d.
(TIF)

Figure S3 **Percent positive response to the blank.** Raw percent response data for mosquitoes responding to the un-scented blank control pipettes during the testing phase of the experiment. Data are presented for male and female *Culex quinquefasciatus* adults conditioned to jasmine odor extract from laboratory colony and field-collected material aged 1–2 d or 3–5 d.
(TIF)

Figure S4 **SPSS 16.0 syntax for full factorial binary logistic regression.** SPSS 16.0 syntax for the full factorial binary logistic regression model using backward stepwise variable selection evaluating mosquito age, sex, source, the amount of time between conditioning and testing and the different tests (target, non-target, or blank) on the response variable. This syntax was modified for model selection as described in the statistical analysis section of the manuscript text.
(TIF)

Video S1 **The response of** ***Culex quinquefasciatus*** **to an artificial sugar source.** Mosquitoes (*Culex quinquefasciatus*) denied access to 10% sucrose for 24 hr were introduced to an artificial sugar source by placing a 10% sucrose solution on a clean sheet of paper under an inverted petri dish. Upon exposure male mosquitoes can be observed probing the paper and walking in search for accessible sugar solution.
(MPG)

Video S2 **The Probing Walking Response (PWR) of mosquitoes to a close artificial sugar source.** Mosquitoes (*Culex quinquefasciatus*) denied access to 10% sucrose solution for 24 hr were allowed to feed upon a green-dyed 10% sucrose solution. Upon exposure the mosquitoes exhibit the Probing Walking Response (PWR) which includes walking, and probing surfaces with the proboscis in search for sugar. Two male mosquitoes can be seen exhibiting this response in the video marked by a yellow and a blue arrow. A resting female can also be observed upon filling with the sugar solution (purple arrow).
(MPG)

Video S3 **The Probing Walking Response (PWR) of mosquitoes on corpses.** Mosquitoes (*Culex quinquefasciatus*) that had been denied access to 10% sucrose solution for 24 hr exhibited the PWR upon corpses in the colony cage prior to introduction of a sucrose solution. The mosquitoes appear to be probing the antennal bases and anterior spiracle which may provide access to any remaining body fluids.
(MPG)

Video S4 **The Probing Walking Response (PWR) of mosquitoes to flowers.** Mosquitoes (*Culex quinquefasciatus*) exposed to a picked *Lantana* sp. flower search for nectar using the same probing walking response observed with respect to artificial sugar sources.
(MPG)

Video S5 **Conditioning of a single mosquito.** A single mosquito (*Culex quinquefasciatus*) is conditioned by offering a glass micropipette coated with the conditioning target odor and filled with a 10% sucrose solution. The mosquito is allowed to feed for 10 sec.
(MPG)

Video S6 **A positive response to conditioning with the Probing Walking Response (PWR).** A single mosquito (*Culex quinquefasciatus*) is tested for response following conditioning and exhibits a positive PWR response to an empty glass micropipette coated with the target odor (jasmine). This response was recorded as a positive response to later analysis. Although gloves were not worn for the purposes of this demonstration, video they were worn at all times during experimental conditioning and testing.
(MPG)

Video S7 **A negative response to conditioning.** A single mosquito (*Culex quinquefasciatus*) is tested for response following conditioning and fails to exhibit the PWR in response to an empty glass micropipette coated with the non-target odor (geraniol). This was recorded as a negative response for later analysis. Negative responses also included a failure to respond to the odor indicated with no visible response. Although gloves were not worn for the purposes of this demonstration, video they were worn at all times during experimental conditioning and testing.
(MPG)

## References

[1] Murlis J, Elkinton JS, Cardé RT (1992). Odor plumes and how insects use them.. Ann Rev Entomol.

[2] Stephens DW, Papaj DR, Lewis AC (1993). Learning and behavioral ecology: incomplete information and environmental predictability.. Insect Learning: Ecological and Evolutionary Perspectives.

[3] Turlings TCJ, Wäckers FL, Vet LEM, Lewis WJ, Tumlinson JH, Papaj DR, Lewis AC (1993). Learning and host-finding cues by hymenopterous parasitoids.. Insect Learning: Ecological and Evolutionary Perspectives.

[4] Dukas R (2008). Evolutionary biology of insect learning.. Ann Rev Entomol.

[5] Vet LEM, Lewis WJ, Cardé RT, Carde RT, Bell WJ (1995). Parasitoid foraging and learning.. Chemical Ecology of Insects.

[6] Tully T, Preat T, Boynton SC, Del Vecchio M (1994). Genetic dissection of consolidated memory in *Drosophila*.. Cell.

[7] Tempel BL, Bonini N, Dawson DR, Quinn WG (1983). Reward learning in normal and mutant *Drosophila*.. PNAS.

[8] Spatz HC, Emanns A, Reichert H (1974). Associative learning of Drosophila melanogaster.. Nature.

[9] Quinn WG, Harris WA, Benzer S (1974). Conditioned behavior in *Drosophila melanogaster*.. PNAS.

[10] Tully T, Quinn WG (1985). Classical conditioning and retention in normal and mutant *Drosophila melanogaster*.. Comp Physiol A.

[11] Tully T (1984). *Drosophila* learning: Behavior to biochemistry.. Behav Gen.

[12] Keene AC, Waddell S (2007). Drosophila olfactory memory: single genes to complex neural circuits.. Nat Rev Neurosci.

[13] McGuire SE, Deshazer M, Davis RL (2005). Thirty years of olfactory learning and memory research in *Drosophila melanogaster*.. Prog Neurobiol.

[14] Bitterman ME, Menzel R, Fietz A, Schafer S (1983). Classical conditioning of proboscis extension reflex in honeybees (*Apis mellifera*).. J Comp Psych.

[15] Meller VH, Davis RL (1996). Biochemistry of insect learning: Lessons from bees and flies.. Insect Biochem Molec Biol.

[16] Gould JL, Papaj DR, Lewis AC (1993). Ethological and comparative perspectives on honey bee learning.. Insect Learning: Ecological and Evolutionary Perspectives.

[17] Daly KC, Smith BH (2000). Associative learning in the moth *Manduca sexta*.. J Exp Biol.

[18] Christensen TA, Mustaparta H, Hildebrand JG (1989). Discrimination of sex pheromone blends in the olfactory system of the moth.. Chem Senses.

[19] Phelan PL, Baker TC (1987). Evolution of male pheromones in moths: Reproductive isolation through sexual selection?. Science.

[20] Meiners T, Wäckers F, Lewis WJ (2002). The effect of molecular structure in olfactory discrimination by the parasitoid *Microplitis croceipes*.. Chem Senses.

[21] Tomberlin JK, Rains GC, Sanford MR (2008). Development of *Microplitis croceipes* as a biological sensor.. Ent Exp Appl.

[22] Rains GC, Utley SL, Lewis WJ (2006). Behavioral monitoring of trained insects for chemical detection.. Biotechnology Progress.

[23] Takasu K, Lewis WJ (1996). The role of learning in adult food location by the larval parasitoid, *Microplitis croceipes* (Hymenoptera: Braconidae).. J Insect Behav.

[24] Wäckers FL, Bonifay C, Lewis WJ (2002). Conditioning of appetitive behavior in the Hymenopteran parasitoid *Microplitis croceipes*.. Ent Exp Appl.

[25] McCall PJ, Kelly DW (2002). Learning and memory in disease vectors.. Trends in Parasit.

[26] Woolhouse MEJ, Dye C, Etard J-F, Smith T, Charlwood JD (1997). Heterogeneities in the transmission of infectious agents: Implications for the design of control programs..

[27] Smith DL, Dushoff J, Snow RW, Hay SI (2005). The entomological inoculation rate and *Plasmodium falciparum* infection in African children.. Nature.

[28] Ribbands CR (1949). Studies on the attractiveness of human populations to Anophelines.. Bull Entomol Res.

[29] Charlwood JD, Graves PM, Marshall TFDC (1988). Evidence for a ‘memorized' home range in *Anopheles farauti* females in Papua New Guinea.. Med Vet Entomol.

[30] McCall PJ, Mosha FW, Njunwa KJ, Sherlock K (2001). Evidence for memorized site-fidelity in *Anopheles arabiensis*.. Trans Roy Soc Trop Med Hyg.

[31] Hii JLK, Chew M, Sang VY, Munstermann LE, Tan SG (1991). Population genetic analysis of host seeking and resting behaviors in the malaria vector, *Anopheles balabacensis* (Diptera: Culicidae).. J Med Entomol.

[32] Mwandawiro C, Boots M, Tuno N, Suwonkerd W, Tsuda Y (2000). Heterogeneity in the host preference of Japanese Encephalitis vectors in Chiang Mai, northern Thailand.. Trans Roy Soc Trop Med Hyg.

[33] Monath TP (1988). The Arboviruses: Epidemiology and Ecology..

[34] Ludlam KW, Jachowski LA, Otto GF (1970). Potential vectors of *Dirofiliaria immitis*.. J Amer Vet Med Assoc.

[35] White GB (1989). Lymphatic Filariasis. Geographic Distribution of Arthropod-borne Diseases and their Principal Vectors..

[36] Foster WA (1995). Mosquito sugar feeding and reproductive energetics.. Annu Rev Entomol.

[37] Alonso WJ, Schuck-Paim C (2006). The ‘ghosts' that pester studies on learning in mosquitoes: guidelines to chase them off.. Med Vet Entomol.

[38] Schlein Y, Müller G (2008). An approach to mosquito control: using dominant attraction of flowering *Tamarix jordanis* tree against *Culex pipiens*.. J Med Entomol.

[39] Müller G, Schlein Y (2006). Sugar questing mosquitoes in arid areas gather on scarce blossoms that can be used for control.. Int J Parasit.

[40] Marshall JM, Taylor CE (2009). Malaria control with transgenic mosquitoes.. PLoS Med.

[41] Coates CJ (2000). A mosquito transformed.. Nature.

[42] Alonso WJ, Wyatt TD, Kelly DW (2003). Are vectors able to learn about their hosts? A case study with *Aedes aegypti* mosquitoes.. Mem Inst Oswaldo Cruz.

[43] Tomberlin JK, Rains GC, Allan SA, Sanford MR, Lewis WJ (2006). Associative learning of odor with food- or blood-meal by *Culex quinquefasciatus* Say (Diptera: Culicidae).. Naturwissen.

[44] Jhumur US, Dotterl S, Jurgens A (2006). Naive and conditioned responses of *Culex pipiens pipiens* biotype *molestus* (Diptera: Culicidae) to flower odors.. J Med Entomol.

[45] Papaj DR, Snell-Rood EC (2007). Memory flies sooner from flies that learn faster.. PNAS.

[46] Mery F, Belay AT, So AKC, Sokolowski MB, Kawecki TJ (2007). Natural polymorphism affecting learning and memory in Drosophila.. Proceedings of the National Academy of Sciences.

[47] Haeger JS (1955). The non-blood feeding habits of *Aedes taeniorhyndus* (Diptera, Culicidae) on Sanibel Island, Florida.. Mosq News.

[48] Sanford MR (2010). Associative Learning Capabilities of Adult *Culex quinquefasciatus* Say and Other Mosquitoes..

[49] Gibson G, Torr SJ (1999). Visual and olfactory responses of haematophagous Diptera to host stimuli.. Med Vet Entomol.

[50] Friend WG, Smith JJB, Tanner RJ (1989). Ingestion and diet destination in *Culiseta inornata*: responses to water, sucrose and cellobiose.. Physiol Entomol.

[51] Telang A, Wells MA (2004). The effect of larval and adult nutrition on successful autogenous egg production by a mosquito.. J Insect Physiol.

[52] Skinner WA, Tong H, Pearson T, Strauss W, Maibach H (1965). Human sweat components attractive to mosquitoes.. Nature.

[53] Skinner WA, Tong H, Johnson H, Maibach H, Skidmore D (1968). Human sweat components - Attractancy and repellency to mosquitoes.. Experientia.

[54] Kramer WL, Mulla MS (1979). Oviposition attractants and repellents of mosquitoes: Oviposition responses of *Culex* mosquitoes to organic infusions.. Environ Entomol.

[55] Yu D, Ponomarev A, Davis RL (2004). Altered representation of the spatial code for odors after olfactory classical conditioning: memory trace formation by synaptic recruitment.. Neuron.

[56] Yuval B (1992). The other habit: sugar feeding by mosquitoes.. Bull Soc Vector Ecol.

[57] Reisen WK, Reeves WC, Reeves WC, Asman SM, Hardy JL, Milby MM, Reisen WK (1990). Chapter VI: Bionomics and Ecology of Culex tarsalis and other potential mosquito vector species.. Epidemiology and Control of Mosquito-borne Arboviruses in California, 1943-1987.

[58] Mery F (2007). Aging and its differential effects on consolidated memory forms in *Drosophila*.. Exp Gerantol.

[59] Tamura T, Chiang A, Ito N, Liu H, Horiuchi J (2003). Aging specifically impairs *amnesiac*-dependent memory in *Drosophila*.. Neuron.

[60] Mery F, Kawecki TJ (2002). Experimental evolution of learning ability in fruit flies.. PNAS.

[61] Pagani MR, Oishi K, Gelb BD, Zhong Y (2009). The phosphatase SHP2 regulates the spacing effeect for long-term memory induction.. Cell.

[62] Hartz SM, Ben-Shahar Y, Tyler M (2001). Logistic growth curve analysis in associative learning data.. Anim Cogn.

[63] DeMaris A (1995). A tutorial in logistic regression.. J Marriage Fam.

[64] Lewis WJ, Tumlinson JH (1988). Host detection by chemically mediated associative learning in a parasitic wasp.. Nature.

[65] Tertuliano M, Olson DM, Rains GC, Lewis WJ (2004). Influence of handling and conditioning protocol on learning and memory of *Microplitis croceipes*.. Ent Exp Appl.

[66] Wäckers F, Bonifay C, Vet LEM, Lewis J (2006). Gustatory response and appetitive learning in *Microplitis croceipes* in relation to sugar type and concentration.. Anim Biol.

[67] Kennedy JS (1978). The concepts of olfactory ‘arrestment' and ‘attraction'.. Physiol Entomol.

[68] Cardé RT (2007). Odour Plumes and Odour-Mediated Flight in Insects: John Wiley & Sons, Ltd.

[69] Reiter P (1986). A standardized procedure for the quantitative surveillance of certain *Culex* mosquitoes by egg raft collection.. J Amer Mosq Control Assoc.

[70] Bowen MF (1992). Terpene-sensitive receptors in female *Culex pipiens* mosquitoes: electrophysiology and behaviour.. J Insect Physiol.

[71] SPSS I (2007). SPSS Graduate Pack 16.0 for Windows..

[72] JMP (1989). Version 7..

