# Supplementary figures and images for: Conditioning Individual Mosquitoes to an Odor: Sex, Source, and Time
Source: PLoS One. 2011 Aug 26;6(8):e24218. doi: 10.1371/journal.pone.0024218 (PMC3162609; doi:10.1371/journal.pone.0024218)

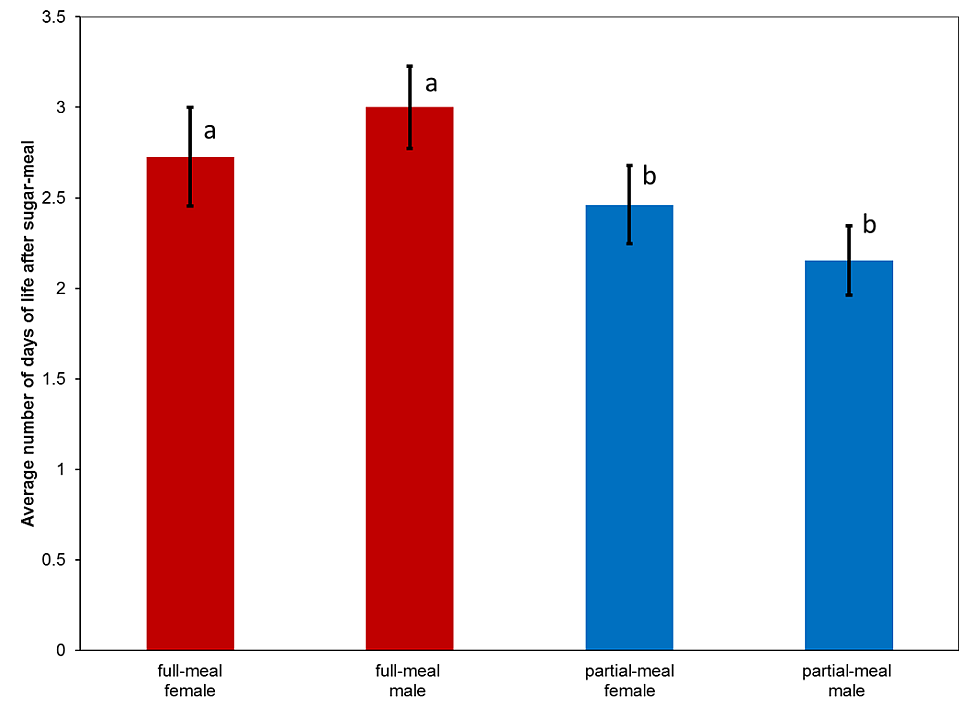

Supplement: Figure S1 — Mosquito lifespan following partial or full sugar-meal. Average number of days of life following either a full sugar-meal or a partial sugar-meal, as would be received following the conditioning protocol, for laboratory colony derived male and female Culex quinquefasciatus. Letters indicate a significant difference between treatments as evaluated with ANOVA at α = 0.05 level. (TIF) [file pone.0024218.s001.tif]

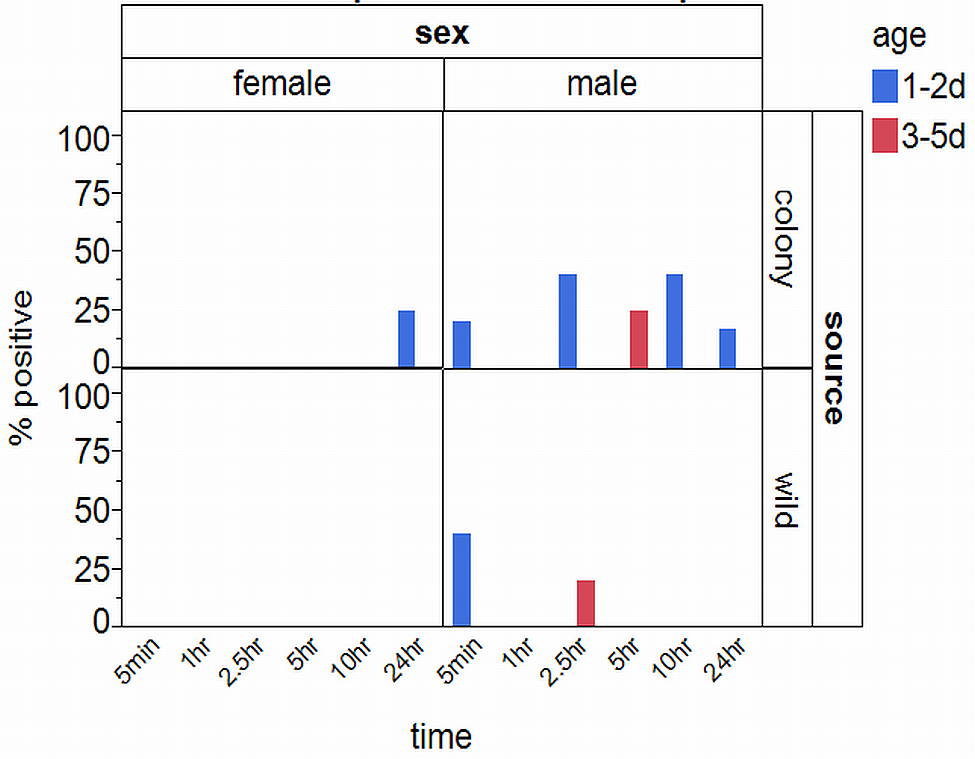

Supplement: Figure S2 — Percent positive response to the non-target odor (geraniol). Raw percent positive response data for mosquitoes tested to the non-target odor of geraniol. Data are presented for male and female Culex quinquefasciatus adults conditioned to jasmine odor extract from laboratory colony and field-collected material aged 1–2 d or 3–5 d. (TIF) [file pone.0024218.s002.tif]

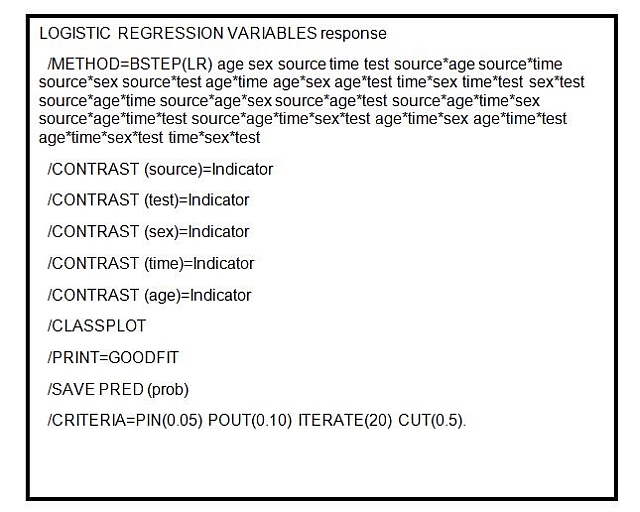

Supplement: Figure S4 — SPSS 16.0 syntax for full factorial binary logistic regression. SPSS 16.0 syntax for the full factorial binary logistic regression model using backward stepwise variable selection evaluating mosquito age, sex, source, the amount of time between conditioning and testing and the different tests (target, non-target, or blank) on the response variable. This syntax was modified for model selection as described in the statistical analysis section of the manuscript text. (TIF) [file pone.0024218.s004.tif]
